# Redefining the Role of ADAM17 in Renal Proximal Tubular Cells and Its Implications in an Obese Mouse Model of Pre-Diabetes

**DOI:** 10.3390/ijms222313093

**Published:** 2021-12-03

**Authors:** Vanesa Palau, Sofia Villanueva, Josué Jarrín, David Benito, Eva Márquez, Eva Rodríguez, María José Soler, Anna Oliveras, Javier Gimeno, Laia Sans, Marta Crespo, Julio Pascual, Clara Barrios, Marta Riera

**Affiliations:** 1 Department of Nephrology, Hospital del Mar-Institut Hospital del Mar d’Investigacions Mèdiques (IMIM), 08003 Barcelona, Spain; vpalau@imim.es (V.P.); villanuevas2c@gmail.com (S.V.); JavierJCarriel@outlook.com (J.J.); david.benito.guasch@gmail.com (D.B.); eva.marquez.mosquera@psmar.cat (E.M.); erodriguezg@psmar.cat (E.R.); aoliveras@psmar.cat (A.O.); lsans@psmar.cat (L.S.); mcrespo@psmar.cat (M.C.); julpascual@gmail.com (J.P.); 2 Nephrology Research Group, Vall d’Hebron Research Institute (VHIR), Nephrology Department, Hospital Universitari Vall d’Hebron, Universitat Autònoma de Barcelona, 08035 Barcelona, Spain; mjsoler01@gmail.com; 3 Department of Pathology, Hospital del Mar, 08003 Barcelona, Spain; JGimenoBeltran@parcdesalutmar.cat

**Keywords:** ADAM17, obesity, pre-diabetes, renal proximal tubular cells

## Abstract

Acute and chronic kidney lesions induce an increase in A Disintegrin And Metalloproteinase domain 17 (ADAM17) that cleaves several transmembrane proteins related to inflammatory and fibrotic pathways. Our group has demonstrated that renal ADAM17 is upregulated in diabetic mice and its inhibition decreases renal inflammation and fibrosis. The purpose of the present study was to analyze how *Adam17* deletion in proximal tubules affects different renal structures in an obese mice model. Tubular *Adam17* knockout male mice and their controls were fed a high-fat diet (HFD) for 22 weeks. Glucose tolerance, urinary albumin-to-creatinine ratio, renal histology, and pro-inflammatory and pro-fibrotic markers were evaluated. Results showed that wild-type mice fed an HFD became obese with glucose intolerance and renal histological alterations mimicking a pre-diabetic condition; consequently, greater glomerular size and mesangial expansion were observed. *Adam17* tubular deletion improved glucose tolerance and protected animals against glomerular injury and prevented podocyte loss in HFD mice. In addition, HFD mice showed more glomerular macrophages and collagen accumulation, which was prevented by *Adam17* deletion. Galectin-3 expression increased in the proximal tubules and glomeruli of HFD mice and ameliorated with *Adam17* deletion. In conclusion, *Adam17* in proximal tubules influences glucose tolerance and participates in the kidney injury in an obese pre-diabetic murine model. The role of ADAM17 in the tubule impacts on glomerular inflammation and fibrosis.

## 1. Introduction

Obesity has become a worldwide pandemic recognized as a major and independent risk factor for the development of chronic kidney disease (CKD), and its prevalence will increase in the next decade [1]. Obesity is characterized by the concurrent existence of glucose intolerance, diabetes, dyslipidemia, and cardiovascular disease [2]. In an obese state, factors derived from adipocytes induce macrophage activation and infiltration. These activated macrophages secrete a large amount of adipokines, pro-inflammatory cytokines, and chemokines into the circulation, which make obesity a chronic inflammation state that leads to the eventual development of insulin resistance [3,4].

Some of the deleterious renal consequences of obesity may be mediated by comorbid factors such as diabetes mellitus or hypertension, but also hyperactivation of endocrine activity in the adipose tissue could impact target organs. These include the development of inflammation, oxidative stress, abnormal lipid metabolism, activation of the renin–angiotensin–aldosterone system, and increased production of insulin and insulin resistance [1,4]. 

A Disintegrin And Metalloproteinases (ADAMs) are membrane-anchored cell surface and secreted proteins [5] that play an important role modulating cell signaling and biological responses by ectodomain shedding of various molecules including cytokines, growth factors, receptors, cell adhesion molecules, and enzymes [5,6]. ADAM17, one of the most studied ADAMs family members, contributes to the development of chronic kidney disease by activating inflammatory and proliferative processes through Tumor Necrosis Factor alpha (TNF-α) and Endothelial Growth Factor Receptor (EGFR) signaling pathways [7]. There is a well-recognized link between TNF-α and obesity, inflammation, and hyperglycemia. In obese and insulin-resistant patients, as well as in animal models, adipose tissue shows an increased expression of TNF-α [8]. Due to the key role of ADAM17 in diverse processes and pathologies, it has been proposed as a target for developing new therapies for the treatment of inflammation and inflammation-associated cancer. Interestingly, blockers of ADAM17 have been considered for clinical trials in several diseases including cancer and type 1 and type 2 diabetes (NCT04630769, NCT02141451, NCT00312780, NCT04557228, NCT01223196).

In experimental studies, high-fat diet (HFD) renders obesity, increased systolic blood pressure, and systemic metabolic alterations related to insulin resistance, increased fasting blood glucose, abnormal lipid levels, and renal alterations such as hyperfiltration with increased albuminuria and progression of glomerulopathies [9]. Interestingly, either a genetic reduction of ADAM17 expression or pharmacological inhibition of ADAM17 in HFD-fed mice partially protected from obesity and insulin resistance compared with wild-type mice [10,11], suggesting that ADAM17 is an important mediator in the development of obesity-induced metabolic disorders.

In healthy kidneys, ADAM17 is mainly expressed in distal tubules, while it is mostly undetected in proximal tubules, mesangium, and endothelial cells. In contrast, in renal diseases, ADAM17 expression appears de novo in proximal tubules, peritubular capillaries, glomerular mesangium, and interstitial inflammatory cells [12]. The specific role of ADAM17 in proximal tubular cells in obese high-fat diet models has not been addressed. For that reason, the present study aims to decipher whether specific *Adam17* deletion on renal proximal tubular cells is able to modulate kidney pathophysiology, inflammatory, and fibrotic processes in an induced obese pre-diabetic mice model fed a high-fat diet. 

## 2. Results

### 2.1. Tubular Adam17 Deletion Modifies Blood Glucose Levels and Renal Function in Obese Mice

To investigate the impact of renal proximal tubular *Adam17* deletion on kidneys from obese mice, we studied male animals fed an HFD for 22 weeks. As shown in Table 1, the HFD groups were heavier as compared to mice fed a standard diet (SD). HFD mice gained 50 g of body weight at the end of the study and were considered obese. This change was not reflected in kidney weight, where all groups showed similar values. 

As depicted in Table 1, fasting blood glucose levels were higher in control mice fed an HFD as compared to mice fed an SD, but without exceeding 250 mg/dL. Therefore, HFD mice were considered pre-diabetic, as described before [13]. Interestingly, HFD mice with proximal tubular *Adam17* deletion showed similar fasting blood glucose levels as compared to SD mice. To test kidney damage, urine albuminuria was calculated by the albumin-to-creatinine ratio (ACR). As shown in Table 1, all HFD-fed mice had a significant increase in ACR.

### 2.2. Glucose Homeostasis Is Influenced by Tubular Adam17 Deletion 

**Figure 1:**
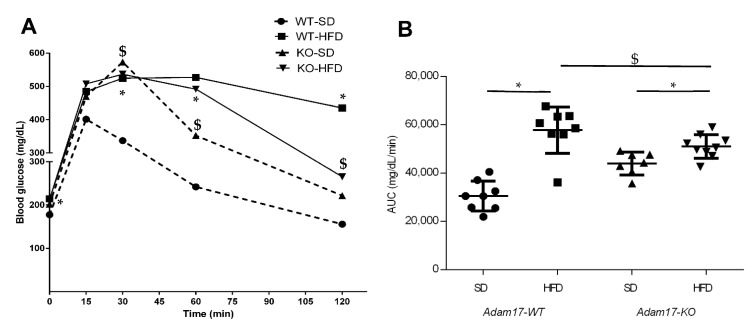
Intraperitoneal glucose tolerance tests performed at 32 weeks of age in male mice. (**A**) Blood glucose levels were measured at 0, 15, 30, 60, and 120 min post glucose bolus injection. (**B**) Area under the curve was calculated. Data are expressed as mean ± SD. Abbreviations: SD, standard diet; HFD, high-fat diet; *Adam17WT*, wild type; *Adam17KO*, knockout. * *p* < 0.05 HFD vs. SD; $ *p* < 0.05 KO vs. WT.

Glucose tolerance was measured, in all groups at 32 weeks of age, by performing an intraperitoneal glucose tolerance test (IPGTT) to evaluate the impact of HFD and proximal tubular *Adam17* deletion in theses mice (Figure 1A). Obese wild-type mice demonstrated significant glucose intolerance compared to the wild-type group fed an SD after 30, 60, and 120 min post injection. Interestingly, obese knockout mice presented lesser glucose intolerance after 120 min post injection as compared to obese wild-type mice. Obese animals with tubular *Adam17* deletion showed higher glucose intolerance compared to knockout animals fed an SD after 60 min post injection. 

The area under the curve (AUC) value was increased in all obese mice fed an HFD compared to control groups (Figure 1B). However, the deletion of *Adam17* in the proximal tubule favored the decrease in AUC value compared to obese wild-type mice. These data suggest the usefulness of our model of HFD-induced obesity in mice to study metabolic syndrome.

**Figure 2:**
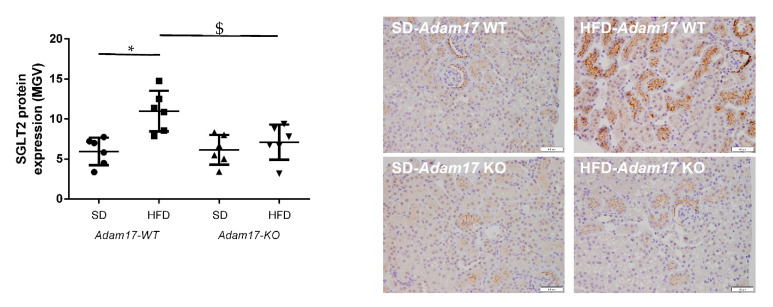
Influence of HFD and proximal tubular *Adam17* deletion on SGLT2 protein expression. Quantification of the SGLT2 immunostaining and representative images depicting SGLT2 staining from all experimental groups. 200× magnification, scale bar 50 μm. Data are expressed as mean ± SD. Abbreviations: SD, standard diet; HFD, high-fat diet; *Adam17WT*, wild type; *Adam17KO*, knockout. * *p* < 0.05 HFD vs. SD, $ *p* < 0.05 KO vs. WT.

Sodium Glucose Co-transporter 2 (SGLT2) was analyzed in kidney samples as the main promoter of glucose reabsorption in the proximal tubule. We found that hyperglycemia increased SGLT2 expression in obese wild-type mice. When this analysis was performed in obese knockout mice, a significant decrease of SGLT2 expression was found as compared to obese wild-type mice (Figure 2).

### 2.3. Renal Histology Is Modified in Obese Tubular Adam17 Knockout Mice

Periodic acid-Schiff (PAS)-stained kidney samples revealed renal injury after 22 weeks of HFD. Obese wild-type mice presented different degrees of global glomerulosclerosis and tubular injury.

**Figure 3:**
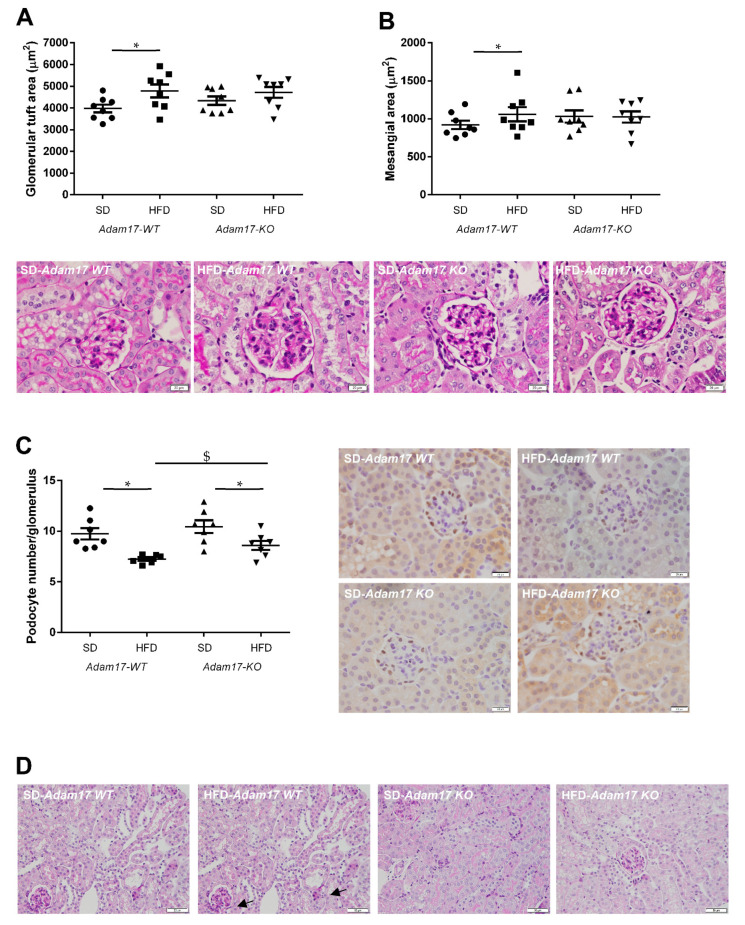
Influence of HFD and proximal tubular *Adam17* deletion on renal structures. (**A**,**B**) Glomerular tuft area and mesangial area values. Representative images depicting PAS staining from all experimental groups. 400× magnification, scale bar 20 μm. (**C**) Glomerular podocyte number represented as the number of positive cells per glomerulus after WT-1 immunostaining. Representative images depicting WT-1 staining from all experimental groups. 400× magnification, scale bar 20 μm. (**D**) Periodic acid-Schiff (PAS)-stained kidney cortex sections from SD- or HFD-fed mice. Black arrows indicate brush border disruption. 200× magnification, scale bar 50 μm. Data are expressed as mean ± SD. Abbreviations: SD, standard diet; HFD, high-fat diet; *Adam17WT*, wild type; *Adam17KO*, knockout. * *p* < 0.05 HFD vs. SD, $ *p* < 0.05 KO vs. WT.

Regarding the glomerular compartment, obese wild-type mice presented a significant increase in glomerular tuft area and also mesangial matrix expansion. This glomerular alterations were attenuated in obese knockout mice, which showed values similar to those obtained for control animals (Figure 3A,B).

The number of podocytes in the glomeruli was significantly decreased in all obese mice. However, *Adam17* deletion significantly prevented that loss observed in wild-type animals with HFD. That finding may indicate that tubular *Adam17* deletion induced a certain degree of protection against podocyte loss (Figure 3C).

Regarding the tubular compartment, proximal tubules showed severe brush border disruption (black arrows), tubular dilation, and acute tubular necrosis in obese wild-type mice. Interestingly, knockout mice fed an HFD presented preserved tubular brush border when compared to obese wild-type mice (Figure 3D).

### 2.4. Adam17 Deletion Modulates Renal Inflammation

To evaluate whether the deletion of *Adam17* in proximal tubular cells protects from renal inflammation, we test the expression of different pro-inflammatory markers at the gene and protein level.

**Figure 4:**
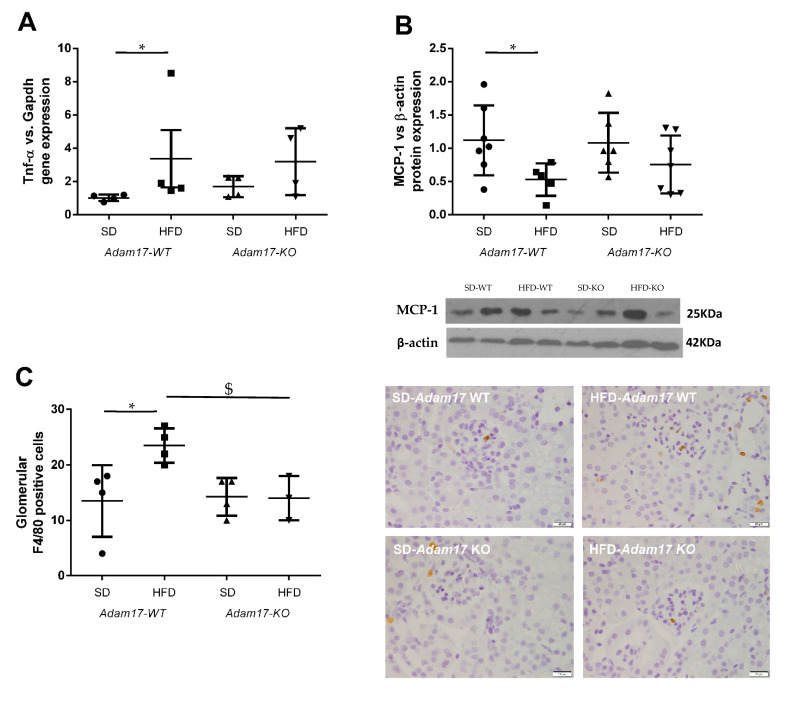
Influence of HFD and proximal tubular *Adam17* deletion on macrophage-mediated inflammation. (**A**) Cortical *Tnf-α* gene expression. (**B**) Cortical MCP-1 protein expression. (**C**) Number of glomerular F4/80 positive cells. Representative images of glomerular F4/80 immunostaining. 400× magnification, scale bar 20 μm. Data are expressed as mean ± SD. Abbreviations: SD, standard diet; HFD, high-fat diet; *Adam17WT*, wild type; *Adam17KO*, knockout. * *p* < 0.05 HFD vs. SD, $ *p* < 0.05 KO vs. WT.

*Tnf-α* gene expression was higher in obese wild-type mice as compared to controls. Obese mice carrying *Adam17* deletion in proximal tubular cells did not show a significant increase on Tnf-α gene expression when comparing with SD knockout mice (Figure 4A). 

Monocyte Chemoattractant Protein-1 (MCP-1) expression in the renal cortex was analyzed by the Western blot technique. This protein has been described as a marker for monocyte chemotaxis. MCP-1 protein expression decreased only in obese wild-type mice (Figure 4B). *Adam17* deletion in obese mice maintained MCP-1 protein levels similarly to the control groups.

To further understand how HFD and *Adam17* deletion modulate renal inflammation, macrophage infiltration was analyzed by F4/80 immunohistochemistry. In contrast to the MCP-1 protein expression, no differences between groups were observed when analyzing interstitial macrophage infiltration (data not shown). As expected, a significant increase on macrophage number was observed into the glomeruli of obese wild-type mice as compared to control. However, obese *Adam17* knockout mice presented a similar number of F4/80^+^ cells to the control groups and a significant decrease when compared to obese wild-type mice (Figure 4C).

### 2.5. Renal Fibrosis Changes with Adam17 Deletion

**Figure 5:**
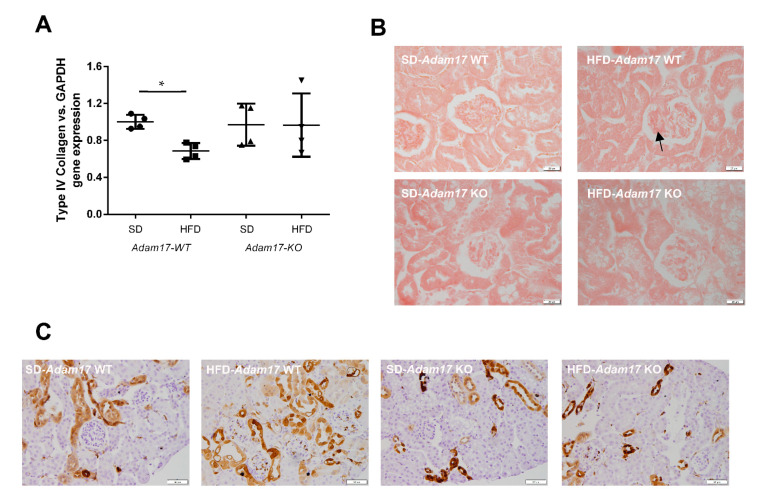
Influence of HFD and proximal tubular *Adam17* deletion on renal fibrotic markers. (**A**) Cortical collagen type IVα1 gene expression. (**B**) Representative images of Sirius Red staining in glomeruli. Black arrow showed collagen I and III accumulation. 400× magnification, scale bar 20 μm. (**C**) Representative images of Gal-3 localization in renal cortex. Black arrow showed glomerular positive Gal-3 cells. 200× magnification, scale bar 50 μm. Data are expressed as mean ± SD. Abbreviations: SD, standard diet; HFD, high-fat diet; *Adam17WT*, wild type; *Adam17KO*, knockout. * *p* < 0.05 HFD vs. SD.

Implication of *Adam17* in renal fibrosis was assessed by gene and protein expression for different fibrotic markers related to the downstream EGFR signaling. In the same line as shown for MCP1, *collagen type IVα1* gene expression was decreased in obese wild-type mice. In contrast, obese knockout mice presented similar levels of *ColIVα1* as the control groups (Figure 5A). 

Glomerular deposition of collagen type I and type III fibers was increased in obese wild-type mice. Conversely, obese mice with tubular *Adam17* deletion exhibited decreased glomerular collagen fibers as compared to wild-type animals (Figure 5B). Since these changes in the collagen deposition were present mainly at the glomerular level, no differences were observed between groups when analyzing cortical collagen (data not shown). 

Galectin-3 (Gal-3) has been linked to the development of renal fibrosis in animal models. To evaluate the influence of *Adam17* deletion on the modulation of Gal-3 expression, an immunohistochemistry technique was performed. As shown in Figure 5C, Gal-3 expression clearly increased in the glomerular endocapillary mononucleated cells, probably monocytes or macrophages, of obese wild-type mice, thus suggesting that these cells may contribute to increase in Gal-3 levels. Moreover, these animals also had positive Gal-3 staining in peritubular capillaries and in convoluted periglomerular tubules. These observations were not found in the SD groups. In obese knockout mice, there was a tendency to decrease Gal-3 expression in both cortical tubules and glomeruli.

### 2.6. Oxidative Stress Induction in the Obese Model with Tubular Adam17 Deletion

**Figure 6:**
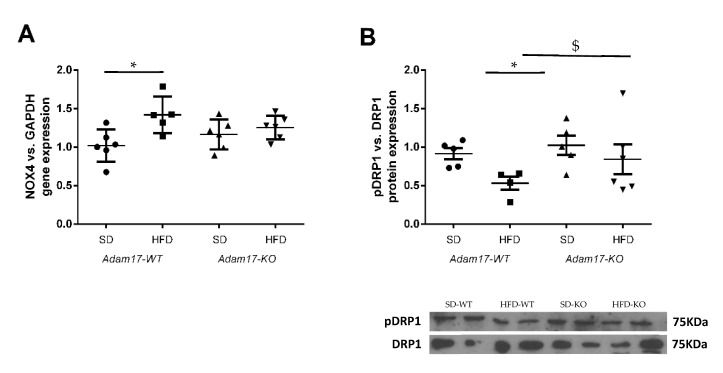
Effect of HFD and proximal tubular *Adam17* deletion on diverse oxidative stress markers. (**A**) Cortical *Nox4* gene expression. (**B**) Cortical pDRP1/DRP1 expression ratio. Data are expressed as mean ± SD. Abbreviations: SD, standard diet; HFD, high-fat diet; *Adam17WT*, wild type; *Adam17KO*, knockout. * *p* < 0.05 HFD vs. SD, $ *p* < 0.05 KO vs. WT.

To explore in more detail the molecular mechanisms involved in renal injury in the obesity mouse model with tubular *Adam17* deletion, we analyzed the gene expression of cortical NADPH oxidase 4 (Nox4) and phosphorylated Dynamin-Related Protein (pDRP1) expression as markers of oxidative stress. As shown in Figure 6A, obese mice increased NOX4 gene expression in the renal cortex. However, the influence of HFD was not observed in *Adam17* knockout mice, as they had similar NOX4 gene expression values compared to controls. 

Thereafter, we analyzed phosphorylated DRP1 expression as a marker of mitochondrial fission in kidney cortex lysates (Figure 6B). Western blot data showed a decrease of pDRP1 expression in the cortex from obese wild-type mice. Interestingly, obese *Adam17* knockout mice presented an increased pDRP1 expression as compared to obese wild-type mice and similar protein levels to those of controls.

## 3. Discussion

ADAM17 expression increases in diabetic kidneys, favoring pro-inflammatory and pro-fibrotic responses [14]. Our group demonstrated in type 1 diabetic mice that *Adam17* deletion in proximal tubular cells protects from diabetic kidney lesions, suggesting a potential therapeutic strategy for the treatment of diabetic nephropathy [15]. The present study sought to evaluate the effect of specific *Adam17* deletion in proximal tubular cells in a mouse model fed a high-fat diet (HFD) for 22 weeks, mimicking a pre-diabetic status. We now demonstrated that ADAM17 in renal tubular cells may modify the systemic inflammatory status reflected in glucose homeostasis, glomerular alterations, tubular fibrosis, and oxidative stress. 

HFD feeding induces many features of the metabolic syndrome including obesity, altered glucose homeostasis, adiposity, insulin resistance, and hyperlipidemia [16]. At the kidney level, HFD feeding leads to albuminuria, glomerulosclerosis, and tubular injury [17]. Our animal model adequately demonstrated the key features of type 2 pre-diabetes including a >40% increase on body weight, higher blood glucose levels, increased albumin-to-creatine ratio, and renal hypertrophy. It is worth mentioning that our HFD animals showed just a discrete but significant increase on the ACR ratio without being considered as severe albuminuria. Regarding glucose homeostasis, obese animals carrying a tubular *Adam17* deletion showed lower levels of blood glucose and improved glucose tolerance. This finding would be explained by two different approaches previously proposed in the literature. One would be the influence of high blood glucose levels in inducing hyperactivation of the sympathetic nervous system and also promoting ADAM17 expression [18]. Matthews et al. hypothesized that an increase in ADAM17 induces renal sympathetic nervous system activity and in turn induces SGLT2 (Sodium Glucose Cotransporter 2) expression, which promotes glucose reabsorption and hyperglycemia [18]. In this line, our study demonstrated that *Adam17* deletion on proximal tubular cells prevents increased blood glucose levels and glucose intolerance to a lesser extent. These results suggested that *Adam17* deletion in proximal tubular cells may decrease SGLT2 activity by hindering glucose reabsorption that favors its excretion in urine. A second approach would be the reduction of systemic insulin resistance by the decrease in chronic inflammation due to the *Adam17* deletion. As previously hypothesized in the adipose tissue, macrophages play an active role in morbid obesity, and macrophage-related inflammatory activities may contribute to the pathogenesis of obesity-induced insulin resistance [4]. Therefore, more studies focused on ADAM17; SGLT2 or chronic inflammation interaction should be performed in more detail in the context of hyperglycemia. 

Marked renal structural changes were seen in obese mice in comparison with the kidneys of lean control animals including global glomerulosclerosis and tubular injury. Regarding glomeruli, previous studies reported the presence of glomerular and mesangial matrix expansion in high-fat diet-fed mice [9,19,20]. Our histological analyses revealed glomerular alterations in obese mice due to an increase in the glomerular tuft area and mesangial expansion. Instead, glomerular hypertrophy and mesangial matrix expansion induced by high blood glucose levels and obesity condition were reduced in *Adam17* knockout mice. These results suggested that the tissue-specific deletion of *Adam17* at proximal tubular cells protected glomeruli from the deleterious effects of a high-fat diet. In this line, Bruneval et al. have described macrophage infiltration in the glomeruli of ApoE knockout mice, which is a model of hyperlipidemia. They presume that macrophage infiltration might be mediated by the local activation of glomerular endothelial cells. These infiltrated macrophages could transform into foam cells, which may contribute to the development of the mesangial expansion observed in ApoE-null mice [21]. In concordance, *Adam17* deletion in proximal tubular cells may slow down mesangial matrix expansion by decreasing glomerular inflammation in obese mice.

Previous studies have demonstrated podocyte loss in HFD-fed mice [22,23]. In the same line, we observed a decrease in podocyte number in the glomeruli of all obese mice. Interestingly, Guo et al. described TNF-α as a key mediator of high-glucose-activated macrophages to induce podocyte apoptosis [24]. This could explain why obese mice with higher blood glucose levels have a decreased number of podocytes. Higher blood glucose levels and hyperlipidemia would lead to the activation of *Adam17* that, in turn, stimulates TNF-α shedding and activation. Blocking renal *Adam17* expression at a proximal tubular level decreased podocyte loss. Probably, this genetic modification might have induced a tubular–glomerular feedback recovery that leads to podocyte protection by blocking TNF-α signaling in obese mice. The decrease in glomerular hypertrophy observed in the *Adam17*-obese KO mice may reflect the restauration of the tubular–glomerular feedback.

In our study, macrophage/monocyte infiltration was observed in the glomeruli of obese mice. F4/80 positive cells were increased in the glomeruli of HFD-fed mice as compared with SD mice. In contrast, no differences were observed when analyzing tubulointerstitial F4/80 immunostaining. These results are in concordance with previous studies that showed glomerular inflammation caused by macrophage infiltration without changes in the tubulointerstitial compartment [25,26]. *Adam17* deletion protected obese mice from glomerular macrophage infiltration, suggesting that the TNF-α signaling pathway is attenuated in *Adam17* knockout proximal tubular cells. 

EGFR hyperactivation by hyperglycemia has also been linked to oxidative stress and fibrosis [27]. More concretely, EGFR-dependent ERK or MAPK signaling mediates TGF-β expression in renal fibrosis, which favors diabetic nephropathy progression through enhancing the synthesis of collagen, fibronectin, and laminin [27,28,29]. To further describe the effect of *Adam17* deletion on obese mice, we analyzed collagen type I and III depositions in different renal compartments. No differences were observed between groups regarding interstitial collagen depositions. In contrast, when focusing at the glomerular level, higher collagen depositions were observed in obese mice, as previously described [27,30]. Interestingly, Fang Q et al. have demonstrated that EGFR inhibition was able to decrease renal fibrosis in HFD-fed mice [27]. In the same line, our results showed that *Adam17* deletion was able to protect the glomeruli of obese mice from collagen depositions, probably by decreasing EGFR signaling. It is worth mentioning that at the gene expression level, collagen type IV α1 was found decreased in obese wild-type mice. This type of collagen participates in the maintenance of the tissue structure [31]. The deletion of *Adam17* returns the expression to basal levels, contributing to reducing the structural changes in the pre-diabetic kidney.

Galectin-3 has been also linked to the development of renal fibrosis in animal models by regulating myofibroblast activation [32,33]. Elevated serum levels of Galectin-3 have been associated with a higher risk of incident CKD and renal dysfunction, suggesting that Galectin-3 can predict renal damage [34]. Kikuchi et al. demonstrated that in normal human kidney, Galectin-3 was detected in the distal tubules but not in the glomeruli. In contrast, Galectine-3-positive cells appear in the glomeruli of patients with diabetic nephropathy. Moreover, they observed that the ratio of Galectin-3-positive cells to the total number of interstitial macrophages was significantly increased during diabetic nephropathy [35]. In agreement with these observations, our SD mice presented a lower cortical expression of Galectin-3 without any positive staining in the glomeruli. In contrast, in obese mice, most glomeruli showed Galectin-3 positive cells. As macrophages have been identified as the major sources of Galectin-3 [36,37], we surmise that the higher macrophage infiltration observed in the glomeruli of the obese mice induced the increased production of Galectin-3 inside the glomerulus. These findings suggest that the Galectin-3 staining pattern could be affected by the HFD-induced glucose alterations. Interestingly, *Adam17* deletion on proximal tubular cells tended to decrease Galectin-3-positive staining in the glomeruli probably by decreasing glomerular inflammation.

Oxidative stress is a key pathogenic factor in diabetes- and obesity-related nephropathy that leads to the development and progression of kidney injury [38,39]. Both mitochondria and NADPH oxidases (Nox) are accepted as major sources of ROS in diabetic nephropathy and chronic kidney disease. Thus, mitochondria-derived oxidative stress has been associated to kidney pro-inflammatory and structural changes in response to lipid overload in obese mice [40]. Moreover, Nox4 is the most abundantly expressed Nox isoform in the kidney, and it has been found to be upregulated and linked to the stimulation of TFG-β and matrix genes, leading to the activation of pro-fibrotic processes associated to kidney fibrosis in diabetic nephropathy [41]. Previous studies have also reported increased *Nox4* gene expression in obese animals [42,43]. In concordance, our results showed increased *Nox4*gene expression in our HFD-fed mice model. Interestingly, this increase in *Nox4*, induced by the high-fat diet, was not observed in *Adam17* knockout mice in the proximal tubular cells. Our results are in line with the previous studies of Wang et al., which demonstrated that TNF-α deletion decreased *Nox4*gene expression in the kidney cortex of obese mice [44]. 

Our results suggest an important role of inflammatory pathways mediated by ADAM17 in the proximal tubular cells. We have demonstrated how this protein impacts on glomeruli and in the tubule. The description of the influence of renal tubular cells modifications in other renal structures highlights the importance of the renal proximal tubular cells in maintaining the tubuloglomerular balance. Concretely, *Adam17* deletion in proximal tubular cells ameliorates glucose homeostasis concomitant with the reduction of SGLT2 and protects against glomerular alterations including glomerular hypertrophy, mesangial expansion, podocyte loss, macrophage infiltration, and fibrosis in an HFD-induced pre-diabetic and obese mice model. Our results abrogate for a partial blocking of the inflammatory and fibrotic processes induced by ADAM17-related pathways as adjuvant treatment for renal pathologies associated to obesity and high glucose levels. Therefore, the blockage of ADAM17 could be a potential therapeutic target for patients with obesity and/or hyperglycemia. 

## 4. Materials and Methods

### 4.1. Animal Experiments

Experiments were performed on wild-type and proximal-tubular *Adam17KO* male mice on C57BL/6 background. Mice were housed in ventilated cages with full access to chow and water. *Adam17* was conditionally deleted in renal proximal tubular cells as previously described by our group [15]. Briefly, the generation of specific proximal tubular knockout mice occurred spontaneously by crossing *Adam17*^flox/flox^ mice (kindly provided by Dr. Raines, Washington University) [45] and spontaneous phosphoenolpyruvate carboxykinase (Pepck)-CreER mice (kindly provided by Dr. Haase, Vanderbilt University) [46]. The Pepck-CreER transgene contains a mutated version of the Pepck promoter, which reduces Pepck expression in the liver by 60% and increases Pepck expression in the kidney by 10-fold [47]. *Adam17*^flox/flox^ mice presented two loxP sites surrounding exon 5 of the *Adam17* gene [45]. Exon 5 was selected for deletion as it encodes for the carboxyl-terminus of the prodomain, upstream of the mature protein, and excision of the sequence results in a frame shift [45].

To induce obesity, at 10 weeks of age, mice were divided randomly into two groups according to diet. Wild-type (*Adam17-WT*) and knockout (*Adam17-KO*) mice were fed a high-fat diet (HFD, 60.3% fat; TD.06414, Envigo, Indianapolis, IN, USA) or standard rodent chow (SD) for controls (7.4% fat; 801151, SDS) during 22 weeks (Supplementary Figure S1). Body weight and fasting blood glucose (BG) from the caudal vein were measured every two weeks until the end of the follow-up. Mice were considered diabetic when BG > 250 mg/dL.

At the end of the follow-up, mice were sacrificed by terminal surgery as previously described [48]. Blood was extracted by cardiac puncture, and serum was obtained by centrifugation at 6000× *g* for 10 min. Mice were perfused with cold PBS prior to kidneys removal and weighting. Half of the right kidney was maintained in 10% formalin solution for paraffin embedding. The remaining tissue was snap frozen on liquid nitrogen and kept at −80 °C for further analyses.

### 4.2. Glucose Tolerance Test

An intraperitoneal glucose tolerance test (IPGTT) was performed 3 days before the end of the study. Mice were fasted for 6 h prior to the test as previously described [49]. Briefly, 2 g/Kg of D-glucose (Sigma-Aldrich, St. Louis, MO, USA) were administrated intraperitoneally, and blood glucose levels were recorded after 15 min, 30 min, 60 min, and 120 min of bolus injection with an Accu-Chek Compact glucometer (Roche, Basel, Switzerland).

### 4.3. Urine Albumin Creatinine Ratio

The albumin-to-creatinine (ACR) ratio was determined on morning spot urine collections obtained on the last week of the follow-up. Urinary albumin levels were measured by ELISA kit (Albuwell M, Exocell, Philadelphia, PA, USA). Creatinine levels were measured by colorimetric assay (Creatinine Companion, Exocell). The albumin-to-creatinine ratio was calculated and expressed as µg Alb/mg Crea [50].

### 4.4. Immunohistochemistry on Paraffined-Embedded Tissue

Paraffin-embedded tissues were cut into 3 µm sections, deparaffined in xylene, and rehydrated through graded alcohols. Sections were stained with periodic acid of Schiff (PAS) for glomerular area and mesangial matrix expansion measurements as previously reported [51]. Then, 20 microphotographs of glomeruli were taken at ×400 magnification for each animal. Image J software was used to analyze glomerular areas.

Immunohistochemistry for podocyte marker Wilms Tumor 1 (WT-1), Galectin-3 (Gal-3), macrophage marker F4/8, and SGLT2 was also performed in sections of paraffin-embedded tissue. Antigen retrieval was carried out with 0.01 M sodium citrate buffer pH6 by heating in a pressure cooker. Endogenous peroxidase was blocked by 3% H_2_O_2_ in TBS1X incubation for 15 min. Non-specific interactions blocking was done by 1% BSA and 3% goat serum for 1 h. Then, sections were incubated with anti-WT-1 antibody (1:1000; sc192, Santa Cruz Biotechnology, Dallas, TX, USA), anti-Galectin-3 antibody (1:1000; 126701, Biolegend, San Diego, CA, USA), anti-F4/80 antibody (1:500; 400501, Biolegend, San Diego, CA, USA), or anti-SGLT2 antibody (1:200; 24654-1-AP, Proteintech, Manchester, UK) at 4 °C overnight. After washing, the slides were incubated with HRP-conjugated anti-rabbit IgG, anti-mouse IgG, or anti-rat IgG during 1 h at room temperature.

Binding of the antibodies was detected by oxidation of DAB using the Liquid DAB + Substrate Chromogen System (Dako, Santa Clara, CA, USA). Samples were counterstained with hematoxylin and dehydrated through graded alcohols and preserved with DPX mounting media (Sigma). Then, 20 microphotographs of glomeruli stained with anti-WT-1 were taken at ×400 magnification. Representative microphotographs of renal cortex stained with anti-SGLT2, anti-Gal-3, or anti-F4/80 were taken at ×200 magnification. 

Picrosirius red staining, as a marker of collagen type I and type III, was performed on 4.5 μm sections of paraffin-embedded kidneys as previously described [52]. Representative images were taken at ×400 magnifications.

### 4.5. Western Blot

Kidney cortical tissue was prepared for immunoblot analysis with antibodies against phosphorylated and total Drp1. Kidney cortex samples were homogenized in extraction buffer containing 50 mM HEPES, pH7.4, 150 mM NaCl, 0.5% Triton X-100, 0.025 mM ZnCl2, 0.1 mM Pefabloc SC Plus (Roche, Basel, Switzerland), EDTA-free protease inhibitor cocktail tablet (Roche, Basel, Switzerland), and phosphatase inhibitor cocktail (Sigma-Aldrich, St. Louis, MO, USA). Protein concentration was determined using the Micro BCA Protein Assay Kit (ThermoFisher Scientific, Waltham, MA, USA). 

Western blot was performed by separating 15 µg of total protein in 7% SDS-polyacrylamide gels and transferring into PVDF membranes (Immobilon-P Millipore). Membranes were incubated in skimmed milk blocking solution (5%) for 1 h and incubated overnight at 4 °C with anti-pDrp1 (Ser616) antibody (1:1000; 4494S, Cell Signaling, Danvers, MA, USA) in 2.5% BSA, anti-Drp1 antibody (5391T, Cell Signaling; 1:1000) in 2.5% BSA, and MCP-1 antibody (1:500; TA336914, Origen, Herford, Germany). HRP-conjugated anti-rabbit IgG antibody and HRP-conjugated anti-mouse IgG antibody were used as secondary antibodies. β-Actin antibody (1:20,000; BS1003, Bioworld, Dublin, Ireland) was used as loading control.

Proteins were detected in films (AGFA CURIX) after 3 min incubation with Clarity Western ECL Substrate (Bio-Rad, Hercules, CA, USA). Protein bands were quantified by densitometry with the ImageJ software.

### 4.6. Gene Expression

Renal cortex RNA was isolated from frozen tissue using the Tripure Isolation Rea-gent (Sigma-Aldrich, St. Louis, MO, USA), as previously reported [53]. First, 1.5 µg of total RNA were retrotranscribed using the High-Capacity cDNA RT Kit (ThermoFisher Scientific, Waltham, MA, USA). Gene expressions for tumor necrosis factor alpha (TNF-α), type IV α1 Collagen, and NADPH Oxidase 4 (Nox4) were determined by Real-Time PCR using LightCycler^®^480 SYBR Green I Master Mix (Roche, Basel, Switzerland). Glyceraldehyde-3-phosphate dehydrogenase (Gapdh) was used as a housekeeping gene. Primer sequences were synthesized by Sigma and described in Supplementary Table S1.

### 4.7. Statistical Analyses

Statistical analyses between groups were performed by one-way ANOVA test (SPSS 22.0 software). Non-parametric Kruskal–Wallis tests were performed between groups. Non-parametric Mann–Whitney tests were used for group-to-group comparisons. Data were expressed as mean ± SD. Significance was defined as *p* < 0.05.

## Figures and Tables

**Table 1:** Fasting blood glucose, body weight, kidney weight, and albumin-to-creatinine ratio were measured after 22 weeks of follow-up. Values are expressed as mean ± SD. Abbreviations: SD, standard diet; HFD, high-fat diet; *Adam17WT*, wild-type; *Adam17KO*, knockout. * *p* < 0.05 HFD vs. SD.

	Fasting Blood Glucose (mg/dL)	Body Weight (g)	Kidney Weight (g)	ACR (μg Alb/mg Crea)
*Adam17WT*—SD	191.91 ± 13.66	34.01 ± 2.70	0.41 ± 0.07	21.80 ± 4.83
*Adam17WT*—HFD	236.63 ± 28.38 *	53.18 ± 1.83 *	0.39 ± 0.04	48.77 ± 21.49 *
*Adam17KO*—SD	199.13 ± 16.80	37.22 ± 4.12	0.39 ± 0.05	26.78 ± 11.71
*Adam17KO*—HFD	218.63 ± 38.53	54.30 ± 8.52 *	0.42 ± 0.08	62.31 ± 27.68 *

## Data Availability

Not applicable.

## Supplementary Materials

The following are available online at https://www.mdpi.com/article/10.3390/ijms222313093/s1.

## References

[1] Kovesdy C.P., Furth S.L., Zoccali C. (2017). Obesity and kidney disease: Hidden consequences of the epidemic. J. Bras. Nefrol..

[2] Kassi E., Pervanidou P., Kaltsas G., Chrousos G. (2011). Metabolic syndrome: Definitions and controversies. BMC Med..

[3] Shoelson S.E., Herrero L., Naaz A. (2007). Obesity, Inflammation, and Insulin Resistance. Gastroenterology.

[4] Xu H., Barnes G.T., Yang Q., Tan G., Yang D., Chou C.J., Sole J., Nichols A., Ross J.S., Tartaglia L.A. (2003). Chronic inflammation in fat plays a crucial role in the development of obesity-related insulin resistance. J. Clin. Investig..

[5] Blobel C.P. (2005). ADAMs: Key components in egfr signalling and development. Nat. Rev. Mol. Cell Biol..

[6] Edwards D.R., Handsley M.M., Pennington C.J. (2009). The ADAM metalloproteinases. Mol. Asp. Med..

[7] Göoz M., Göoz M. (2012). ADAM Proteases as Novel Therapeutic Targets in Chronic Kidney Disease. Chronic Kidney Disease.

[8] Menghini R., Fiorentino L., Casagrande V., Lauro R., Federici M. (2013). The role of ADAM17 in metabolic inflammation. Atherosclerosis.

[9] Deji N., Kume S., Araki S.I., Soumura M., Sugimoto T., Isshiki K., Chin-Kanasaki M., Sakaguchi M., Koya D., Haneda M. (2009). Structural and functional changes in the kidneys of high-fat diet-induced obese mice. Am. J. Physiol. Ren. Physiol..

[10] Serino M., Menghini R., Fiorentino L., Amoruso R., Mauriello A., Lauro D., Sbraccia P., Hribal M.L., Lauro R., Federici M. (2007). Mice heterozygous for tumor necrosis factor-α converting enzyme are protected from obesity-induced insulin resistance and diabetes. Diabetes.

[11] Kaneko H., Anzai T., Horiuchi K., Morimoto K., Anzai A., Nagai T., Sugano Y., Maekawa Y., Itoh H., Yoshikawa T. (2011). Tumor necrosis factor-α converting enzyme inactivation ameliorates high-fat diet-induced insulin resistance and altered energy homeostasis. Circ. J..

[12] Melenhorst W.B., Visser L., Timmer A., van den Heuvel M.C., Stegeman C.A., van Goor H. (2009). ADAM17 upregulation in human renal disease: A role in modulating TGF-α availability?. Am. J. Physiol. Physiol..

[13] Yao M., Li L., Huang M., Tan Y., Shang Y., Meng X., Pang Y., Xu H., Zhao X., Lei W. (2021). Sanye Tablet Ameliorates Insulin Resistance and Dysregulated Lipid Metabolism in High-Fat Diet-Induced Obese Mice. Front. Pharmacol..

[14] Palau V., Pascual J., Soler M.J., Riera M. (2019). Role of ADAM17 in kidney disease. Am. J. Physiol. Physiol..

[15] Palau V., Nugraha B., Benito D., Pascual J., Emmert M.Y., Hoerstrup S.P., Riera M., Soler M.J. (2021). Both specific endothelial and proximal tubular ADAM17 deletion protect against diabetic nephropathy. Int. J. Mol. Sci..

[16] He M.Q., Wang J.Y., Wang Y., Sui J., Zhang M., Ding X., Zhao Y., Chen Z.Y., Ren X.X., Shi B.Y. (2020). High-fat diet-induced adipose tissue expansion occurs prior to insulin resistance in C57BL/6J mice. Chronic Dis. Transl. Med..

[17] Sun Y., Ge X., Li X., He J., Wei X., Du J., Sun J., Li X., Xun Z., Liu W. (2020). High-fat diet promotes renal injury by inducing oxidative stress and mitochondrial dysfunction. Cell Death Dis..

[18] Matthews J., Villescas S., Herat L., Schlaich M., Matthews V. (2021). Implications of ADAM17 activation for hyperglycaemia, obesity and type 2 diabetes. Biosci. Rep..

[19] Declèves A.E., Mathew A.V., Cunard R., Sharma K. (2011). AMPK mediates the initiation of kidney disease induced by a high-fat diet. J. Am. Soc. Nephrol..

[20] Wei P., Lane P.H., Lane J.T., Padanilam B.J., Sansom S.C. (2004). Glomerular structural and functional changes in a high-fat diet mouse model of early-stage Type 2 diabetes. Diabetologia.

[21] Bruneval P., Bariéty J., Bélair M.F., Mandet C., Heudes D., Nicoletti A. (2002). Mesangial expansion associated with glomerular endothelial cell activation and macrophage recruitment is developing in hyperlipidaemic apoE null mice. Nephrol. Dial. Transplant..

[22] Shevalye H., Lupachyk S., Watcho P., Stavniichuk R., Khazim K., Abboud H.E., Obrosova I.G. (2012). Prediabetic nephropathy as an early consequence of the high-calorie/high- fat diet: Relation to oxidative stress. Endocrinology.

[23] Boini K.M., Xia M., Abais J.M., Li G., Pitzer A.L., Gehr T.W.B., Zhang Y., Li P.L. (2014). Activation of inflammasomes in podocyte injury of mice on the high fat diet: Effects of ASC gene deletion and silencing. Biochim. Biophys. Acta Mol. Cell Res..

[24] Guo Y., Song Z., Zhou M., Yang Y., Zhao Y., Liu B., Zhang X. (2017). Infiltrating macrophages in diabetic nephropathy promote podocytes apoptosis via TNF-α-ROS-p38MAPK pathway. Oncotarget.

[25] Lee S.J., Kang J.S., Kim H.M., Lee E.S., Lee J.H., Chung C.H., Lee E.Y. (2019). CCR2 knockout ameliorates obesity-induced kidney injury through inhibiting oxidative stress and ER stress. PLoS ONE.

[26] Kim D.H., Chun S.Y., Lee E.H., Kim B., Yoon B.H., Gil H., Han M.H., Ha Y.S., Lee J.N., Kwon T.G. (2021). IL-10 Deficiency Aggravates Renal Inflammation, Fibrosis and Functional Failure in High-Fat Dieted Obese Mice. Tissue Eng. Regen. Med..

[27] Fang Q., Zou C., Zhong P., Lin F., Li W., Wang L., Zhang Y., Zheng C., Wang Y., Li X. (2016). EGFR mediates hyperlipidemia-induced renal injury via regulating inflammation and oxidative stress: The detrimental role and mechanism of EGFR activation. Oncotarget.

[28] McLennan S.V., Fisher E., Martell S.Y., Death A.K., Williams P.F., Lyons J.G., Yue D.K. (2000). Effects of glucose on matrix metalloproteinase and plasmin activities in mesangial cells: Possible role in diabetic nephropathy. Kidney Int. Suppl..

[29] Ayo S.H., Radnik R.A., Garoni J.A., Glass W.F., Kreisberg J.I. (1990). High glucose causes an increase in extracellular matrix proteins in cultured mesangial cells. Am. J. Pathol..

[30] Xu H., Ma Z., Lu S., Li R., Lyu L., Ding L., Lu Q. (2017). Renal resistive index as a novel indicator for renal complications in high-fat diet-fed mice. Kidney Blood Press. Res..

[31] Zhu D., Kim Y., Steffes M.W., Groppoli T.J., Butkowski R.J., Mauer S.M. (1994). Glomerular distribution of type IV collagen in diabetes by high resolution quantitative immunochemistry. Kidney Int..

[32] Chen S.C., Kuo P.L. (2016). The role of galectin-3 in the kidneys. Int. J. Mol. Sci..

[33] Henderson N.C., Mackinnon A.C., Farnworth S.L., Kipari T., Haslett C., Iredale J.P., Liu F.T., Hughes J., Sethi T. (2008). Galectin-3 expression and secretion links macrophages to the promotion of renal fibrosis. Am. J. Pathol..

[34] Hara A., Niwa M., Noguchi K., Kanayama T., Niwa A., Matsuo M., Hatano Y., Tomita H. (2020). Galectin-3 as a next-generation biomarker for detecting early stage of various diseases. Biomolecules.

[35] Kikuchi Y., Kobayashi S., Hemmi N., Ikee R., Hyodo N., Saigusa T., Namikoshi T., Yamada M., Suzuki S., Miura S. (2004). Galectin-3-positive cell infiltration in human diabetic nephropathy. Nephrol. Dial. Transplant..

[36] Farhad M., Rolig A.S., Redmond W.L. (2018). The role of Galectin-3 in modulating tumor growth and immunosuppression within the tumor microenvironment. Oncoimmunology.

[37] Okamura D.M., Pasichnyk K., Lopez-Guisa J.M., Collins S., Hsu D.K., Liu F.T., Eddy A.A. (2011). Galectin-3 preserves renal tubules and modulates extracellular matrix remodeling in progressive fibrosis. Am. J. Physiol. Ren. Physiol..

[38] Forbes J.M., Coughlan M.T., Cooper M.E. (2008). Oxidative stress as a major culprit in kidney disease in diabetes. Diabetes.

[39] Sharma K. (2016). Obesity and Diabetic Kidney Disease: Role of Oxidant Stress and Redox Balance. Antioxid. Redox Signal..

[40] Ruggiero C., Ehrenshaft M., Cleland E., Stadler K. (2011). High-fat diet induces an initial adaptation of mitochondrial bioenergetics in the kidney despite evident oxidative stress and mitochondrial ROS production. Am. J. Physiol. Endocrinol. Metab..

[41] Muñoz M., López-Oliva M.E., Rodríguez C., Martínez M.P., Sáenz-Medina J., Sánchez A., Climent B., Benedito S., García-Sacristán A., Rivera L. (2020). Differential contribution of Nox1, Nox2 and Nox4 to kidney vascular oxidative stress and endothelial dysfunction in obesity. Redox Biol..

[42] Zhang S.Q., Sun Y.T., Xu T.H., Zhang X.F., Liu Y.Z., Ma J.F., Wang L.N., Yao L. (2014). Protective effect of metformin on renal injury of C57BL/6J mouse treated with high fat diet. Pharmazie.

[43] Jiang F., Lim H.K., Morris M.J., Prior L., Velkoska E., Wu X., Dusting G.J. (2011). Systemic upregulation of NADPH oxidase in diet-induced obesity in rats. Redox Rep..

[44] Gai Z., Kullak-Ublick G.A. (2017). TNF-α Deficiency Prevents Renal Inflammation and Oxidative Stress in Obese Mice. Kidney Blood Press. Res..

[45] Wilson C.L., Gough P.J., Chang C.A., Chan C.K., Frey J.M., Liu Y., Braun K.R., Chin M.T., Wight T.N., Raines E.W. (2013). Endothelial deletion of ADAM17 in mice results in defective remodeling of the semilunar valves and cardiac dysfunction in adults. Mech. Dev..

[46] Rankin E.B., Tomaszewski J.E., Haase V.H. (2006). Renal cyst development in mice with conditional inactivation of the von Hippel-Lindau tumor suppressor. Cancer Res..

[47] Patel Y.M., Yun J.S., Liu J., McGrane M.M., Hanson R.W. (1994). An analysis of regulatory elements in the phosphoenolpyruvate carboxykinase (GTP) gene which are responsible for its tissue-specific expression and metabolic control in transgenic mice. J. Biol. Chem..

[48] Clotet S., Soler M.J., Rebull M., Gimeno J., Gurley S.B., Pascual J., Riera M. (2016). Gonadectomy prevents the increase in blood pressure and glomerular injury in angiotensin-converting enzyme 2 knockout diabetic male mice. Effects on renin-angiotensin system. J. Hypertens..

[49] Roca-Ho H., Palau V., Gimeno J., Pascual J., Soler M.J., Riera M. (2020). Angiotensin-converting enzyme 2 influences pancreatic and renal function in diabetic mice. Lab. Investig..

[50] Riera M., Anguiano L., Clotet S., Roca-Ho H., Rebull M., Pascual J., Soler M.J. (2016). Paricalcitol modulates ACE2 shedding and renal ADAM17 in NOD mice beyond proteinuria. Am. J. Physiol. Physiol..

[51] Riera M., Márquez E., Clotet S., Gimeno J., Roca-Ho H., Lloreta J., Juanpere N., Batlle D., Pascual J., Soler M.J. (2014). Effect of insulin on ACE2 activity and kidney function in the non-obese diabetic mouse. PLoS ONE.

[52] Junqueira L.C.U., Bignolas G., Brentani R.R. (1979). Picrosirius staining plus polarization microscopy, a specific method for collagen detection in tissue sections. Histochem. J..

[53] Clotet-Freixas S., Soler M.J., Palau V., Anguiano L., Gimeno J., Konvalinka A., Pascual J., Riera M. (2018). Sex dimorphism in ANGII-mediated crosstalk between ACE2 and ACE in diabetic nephropathy. Lab. Investig..

