# Supplementary material for: Redefining the Role of ADAM17 in Renal Proximal Tubular Cells and Its Implications in an Obese Mouse Model of Pre-Diabetes
Source: Int J Mol Sci. 2021 Dec 3;22(23):13093. doi: 10.3390/ijms222313093 (PMC8657896; doi:10.3390/ijms222313093)
Supplement: Supplementary file 1 [file ijms-22-13093-s001.zip › ijms-1462784-supplementary.pdf]

## Supplementary Figure S1

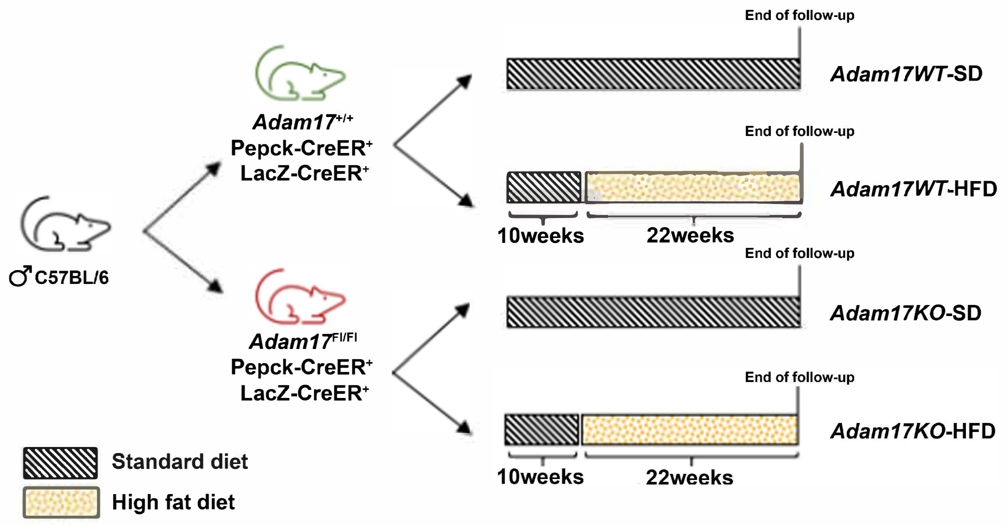

**Supplementary Table S1. Primer sequences used for Real Time qPCR analysis**

| | FORWARD | REVERSE |
|---|---|---|
| **Tnf-α** | 5'-GAC TAG CCA GGA GGG AGA ACA G-3' | 5'-CAG TGA GTG AAA GGG ACA GAA CCT-3' |
| **Col IV** | 5'-TGT CCA TGG CAC CCA TCT CT-3' | 5'-CAC AAA CCG CAC ACC TGC TA-3' |
| **NOX4** | 5'- CTT GGT GAA TGC CCT CAA CT-3' | 5'-TTC TGG GAT CCT CAT TCT GG-3' |
| **GAPDH** | 5'-TCA TTG ACC TCA ACT ACA TGG TCT-3' | 5'-CTT GAC TGT GCC GTT GAA TTT-3' |
